# Down-Regulation of the Longevity-Associated Protein SIRT1 in Peripheral Blood Mononuclear Cells of Treated HIV Patients

**DOI:** 10.3390/cells11030348

**Published:** 2022-01-20

**Authors:** Aleksandra Gruevska, Ángela B. Moragrega, María J. Galindo, Juan V. Esplugues, Ana Blas-García, Nadezda Apostolova

**Affiliations:** 1 Departamento de Farmacología, Universidad de Valencia, 46010 Valencia, Spain; agruevs@alumni.uv.es (A.G.); anbemoes@alumni.uv.es (Á.B.M.); juan.v.esplugues@uv.es (J.V.E.); 2 Fundación para el Fomento de la Investigación Sanitaria y Biomédica de la Comunitat Valenciana (FISABIO), 46020 Valencia, Spain; ana.blas@uv.es; 3 Unidad de Enfermedades Infecciosas—Medicina Interna, Hospital Clínico Universitario de Valencia, 46010 Valencia, Spain; galindo.pepa1@gmail.com; 4 Centro de Investigación Biomédica en Red de Enfermedades Hepáticas y Digestivas (CIBERehd), 46010 Valencia, Spain; 5 Departamento de Fisiología, Universidad de Valencia, 46010 Valencia, Spain

**Keywords:** SIRT1, antiretroviral drugs, HIV, aging, inflammation, PBMC

## Abstract

The activity of sirtuin 1 (SIRT1), a class III histone deacetylase with a critical role in several biological functions, decreases with age and its deficiency is associated with many inflammatory and age-related diseases. It also regulates the chronic immune activation and viral latency during an HIV infection. The life-span and particularly the health span of HIV patients are substantially shortened; however, the participation of SIRT1 in these effects is not clear. We performed a prospective cross-sectional monocentric study that included 70 HIV-infected patients and 43 BMI-, age- and sex-matched uninfected individuals. We found that in the PBMCs of the HIV patients, *SIRT1* mRNA levels were significantly lower (*p* < 0.0001). This decrease, which was corroborated at the protein level, occurred irrespectively of the antiretroviral regimen these patients received and was not significantly related to the general, HIV-related or comorbidity-related parameters. The levels of the major mitochondrial sirtuin *SIRT3* were not altered. Moreover, the strong correlations of *SIRT1* with the leukocyte markers *CD8A* and *CD19* present in the uninfected individuals were absent in the HIV patients. In conclusion, this study showed that the PBMCs of the HIV patients displayed diminished SIRT1 levels and altered correlations of SIRT1 with markers of CD8^+^ T cells and B cells, findings which may be relevant for understanding the complex pathogenic milieu in HIV patients.

## 1. Introduction

Due to the effectiveness and safety of combined antiretroviral therapy (cART), HIV infection has become a manageable, life-long disease [1]. However, HIV patients develop non-AIDS-associated age-linked pathologies at an accelerated rate [2] and their life-span is shorter than that of the general population [3]. By 2030, it is estimated that 75% of HIV patients will be over the age of 50 and more than 80% will have at least one age-related disease [4]. The reasons for the premature aging observed in HIV patients are not fully understood, but abundant evidence points to the presence of chronic low-level inflammation and immune dysfunction, with the cumulative adverse effects of anti-HIV drugs being a contributing factor [5].

Sirtuins are a subject of increasing attention due to their protective roles in various pathophysiological processes, including aging, neurodegeneration, obesity, heart disease, inflammation and cancer [6]. These class III NAD^+^-dependent histone/protein deacetylases include seven subtypes in mammals; among them are SIRT1 and SIRT3, which have the strongest deacetylase activity. The activity of SIRT1, the most extensively studied sirtuin, decreases with increasing age and its deficiency is associated with many inflammatory and age-related diseases, including metabolic syndrome and cardiovascular diseases. SIRT1 deacetylates key histones and multiple non-histone proteins, including p53, forkhead box protein O 1 and 3 (FOXO1/3), peroxisome proliferator activated receptor-gamma coactivator-1 alpha (PGC-1α) and nuclear factor kappa B (NF-κB), and thereby plays a crucial role in transcription regulation [7] and in influencing numerous vital signaling pathways and cell responses [8]. Besides its widely studied role as a metabolic sensor and regulator, more recently SIRT1 has been acknowledged as a key modulator of the inflammatory reactions and the immune response, including both the innate and the acquired components. It possesses anti-inflammatory functions primarily by inhibiting the transcriptional activities of pro-inflammatory factors through deacetylation and also regulates immune cell differentiation in a cell-type-specific manner. For example, the transcription factor FOXO1, which is involved in the transcriptional reprogramming of CD8^+^ T cells, has been identified as an SIRT1 target. At a reduced SIRT1 level, FOXO1 was found to be degraded and the CD8^+^ T cells had enhanced glycolytic and cytotoxic capacities, resulting in immune dysfunction [9].

Importantly, there is a complex and ambiguous interplay between HIV-1 and SIRT1. SIRT1 is a transcriptional coactivator of HIV-1 as it recycles Tat, a protein fundamental to viral gene expression [10], while Tat itself downregulates SIRT1 [11]. Leukocytes are widely considered to reflect systemic diseases. In the present study, we aimed to analyze the levels of SIRT1 in the peripheral blood mononuclear cells (PBMCs) from a heterogenous population of HIV patients in comparison to those of uninfected matched subjects. We also assessed whether *SIRT1* expression was correlated with specific characteristics of HIV patients, including the antiretroviral regimen they were prescribed, and its correlation with markers of specific lymphocyte populations.

## 2. Materials and Methods

### 2.1. Study Design, Subjects and Ethics Statements

This observational cross-sectional study involved 70 HIV-infected men and women (≥18 years of age) recruited at the Hospital Clínico Universitario de Valencia, Valencia, Spain (September 2017–September 2018) who were virologically suppressed via cART (HIV RNA load <40 copies/mL) for at least 6 months at the time of recruitment and 43 age-, sex- and BMI-matched HIV-negative control subjects (volunteer blood donors). Our cohort of patients was chosen to be representative of the currently treated adult HIV-infected population in our community, for which very few exclusion criteria were established (individuals under 18 years of age, pregnant women, newly diagnosed patients and patients whose antiretroviral regimen had been changed in the last 6 months). The study conformed to the principles of the Declaration of Helsinki and the Good Clinical Practice Guidelines, its objectives and procedures were approved by the local investigational review board and all participants provided written informed consent. HIV-infection-related plasma parameters and general biochemical variables (lipids, glucose, transaminases, ferritin and CRP) were evaluated in the Hospital Clínico Universitario de Valencia by routine procedures.

### 2.2. Isolation of PBMCs

PBMCs were isolated by density gradient from venous whole blood (25 mL) with sodium citrate as an anticoagulant, as previously reported [12].

### 2.3. Quantitative RT-PCR

RNA was isolated with the illustra^®^RNAspin Mini RNA Isolation Kit (GE Healthcare, Chicago, IL, USA). Complementary DNA (cDNA) was synthetized using 1 μg of total RNA and the “PrimeScriptTM RT Reagent Kit” (Takara Bio, Kusatsu, Shiga, Japan) in a 20 μL final volume and the presence of 4 μL PrimeScript Buffer, 1 μL PrimeScript RT Enzyme Mix I, 1 μL Oligo dT primer and 1 μL random 6-mers using a SimpliAmp™ Thermal Cycler (Applied Biosystems™, Waltham, MA, USA) under the following conditions: 37 °C (15 min), 85 °C (5 s) and 4 °C (∞). The RT-qPCR was performed with SYBR^®^ Premix Ex Taq™ (Tli RNaseH Plus) (TaKaRa Bio Inc.) containing Ex Taq HS, dNTP mixture, Mg^2+^, Tli RNase H and SYBR Green I, by mixing 5 μL with 1 μL cDNA, 0.2 μM primers (forward and reverse, synthetized by Metabion (Planegg, BY, Germany) or IDT^®^, (Integrated DNA Technologies, Coralville, IA, USA) shown in Table 1) and RNase-free water (10 μL final volume). Reactions were performed using Lightcycler^®^ 96 Real-Time PCR System (Roche Life Science, Penzberg, Germany). Conditions: preincubation for 30 s at 95 °C, 45 cycles of amplification (95 °C for 5 s, 60 °C for 20 s) and lastly 95 °C for 1 s, 65 °C for 15 s, 95 °C for 1 s and 40 °C for 30 s. The number of amplified copies was quantified using standard curves derived from previously purified PCR product (DNA) for each primer pair. Data were normalized using *GAPDH* as a housekeeping gene.

### 2.4. Protein Expression Analysis by Western Blot

Cells were lysed (PhosphoSafe™ Extraction Reagent, EMD Millipore Corp., supplemented with 10× “Complete Mini” protease inhibitor cocktail, Roche Diagnostics GmbH), vortexed (15 s), incubated (5 min at RT) and centrifuged (4 °C 5 min, 16,000 rpm). The protein content was quantified (BCA assay, Pierce™ BCA Protein Assay Kit, Thermo Fisher Scientific, Waltham, MA, USA) and 30 μg of each whole-cell extract was immunoblotted. Primary antibodies: rabbit polyclonal anti-SIRT1 (1:1000, Merck Milipore, Darmstadt, Germany), mouse monoclonal anti-acetyl-histone H3 (H3K9) (Ac-Lys9, 1:1000, Sigma-Aldrich, Stenheim, Germany) and anti-GAPDH (1:15,000, Sigma-Aldrich); secondary antibodies: anti-rabbit (1:5000, Vector laboratories, Burlingame, CA, USA) and anti-mouse (1:2000, Thermo Fisher). Immunolabeling was detected by enhanced chemiluminescence (Luminata™ Crescendo Western HRP substrate, Merck or SuperSignal™ West Femto Maximum Sensitivity Substrate, Thermo Fisher Scientific), and visualized with a digital luminescent image analyzer LAS-3000 Imager (Fujifilm, Tokyo, Japan). Densitometric analysis was performed using Multi Gauge V3.0 software (Fujifilm).

### 2.5. Statistical Analysis and Presentation of Data

Data were analyzed using GraphPad Prism^®^ V6.01 (GraphPad Software, San Diego, CA, USA) and are displayed as median values (quoted with the interquartile range, IQR) or as mean ± standard error of the mean (SEM); normality was assessed with the D’Agostino and Pearson omnibus normality test. Parametric and non-parametric variables were assessed by Student’s unpaired *t*-test and the Mann–Whitney U test, respectively. Correlations are expressed as Pearson’s correlation coefficients (r_p_) when both variables were normally distributed (parametric data) and as Spearman’s correlation coefficients (r_s_) for non-parametric data. Correlation coefficients >0.3 were considered to present positive correlations (the range of 0.3–0.5 and that of 0.5–0.7 were considered weak and moderate positive correlations, respectively) [13]. Significance was inferred from a *p* value < 0.05.

## 3. Results

This study involved 70 HIV-infected men and women (≥18 years of age) and 43 age-, sex- and BMI-matched HIV-negative control subjects whose characteristics are shown in Table 2 and Table 3, respectively.

**Figure 1:**
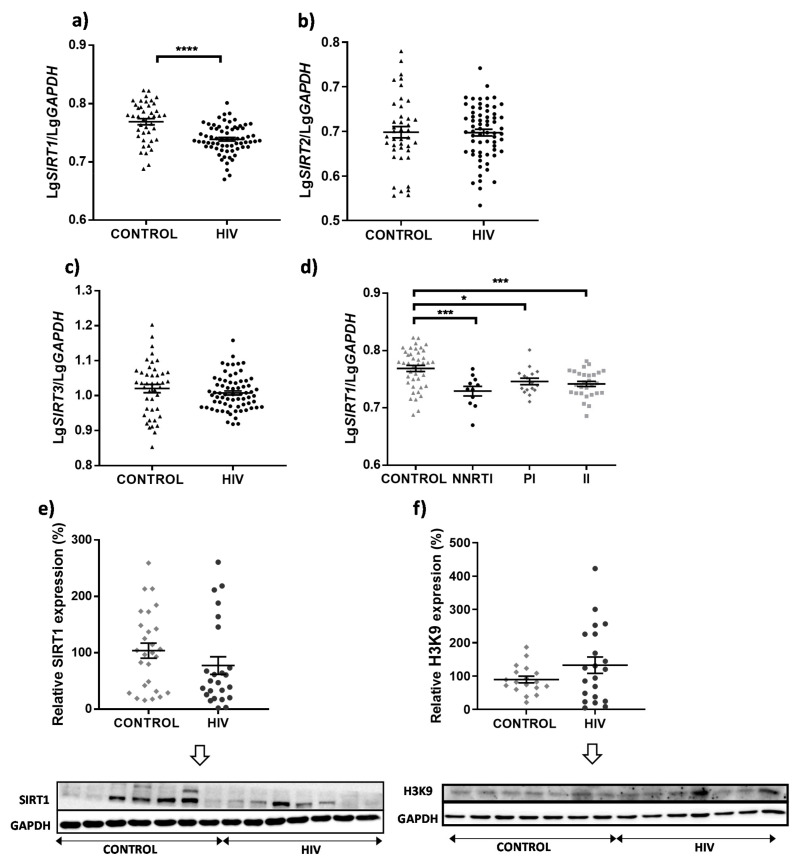
Expression levels of *SIRT1*, *SIRT2*, *SIRT3,* and protein abundance of SIRT1 and acetylated histone 3 in PBMCs obtained from HIV patients and uninfected controls. *SIRT1* (**a**), *SIRT2* (**b**) and *SIRT3* (**c**) mRNA levels in PBMCs of control (*n* = 41–43) and HIV subjects (*n* = 65–69) subjects, determined by RT-qPCR. (**d**) Differences in *SIRT1* expression in PBMCs between controls and HIV patients grouped considering present cART. While all patients had NRTI as the backbone, they differed in the additional drugs in their cART (NNRTI, PI or II). Data (mean ± SEM; for control, *n* = 42; for NNRTIs, *n* = 11; for PIs, *n* = 16; for IIs, *n* = 28) for gene expression were calculated as number of copies of the gene of interest normalized with the number of copies of the housekeeping gene (*GAPDH)*. (**e**,**f**) Western blot analysis using whole-cell protein extracts for SIRT1 and acetylated histone 3 (H3K9). Representative images are shown and graphical representations of the quantified data (mean ± SEM; calculated as % of the control, i.e., the mean value of the protein expression in control group was considered 100%) for *n* = 27 and *n* = 24 in the controls and HIV patients, respectively (for SIRT1) and *n* = 18 and *n* = 21 in the controls and HIV patients, respectively (for H3K9). GAPDH was used as the loading control. Statistical analysis between groups: Student unpaired *t*-test or one-way ANOVA followed by a multiple comparison test, the Bonferroni post-test (* *p* < 0.05, *** *p* < 0.001 **** *p* < 0.0001 vs. control). II, integrase inhibitor; NNRTI, non-nucleoside reverse transcriptase inhibitor; PI, protease inhibitor.

The expression of *SIRT1* in the PBMCs of the HIV patients was significantly diminished compared to that of the uninfected controls (*p* < 0.0001) (Figure 1a), while no differences in the cytoplasmic *SIRT2* or the major mitochondrial sirtuin *SIRT3* were detected (*p* = 0.9528 and *p* = 0.2851, respectively) (Figure 1b,c). We also found that while the control individuals showed a weak albeit significant positive correlation in *SIRT1/SIRT3* expression (r_p_ = 0.374, *p* = 0.014), it was absent in the HIV patients (r_p_ = 0.204, *p* = 0.096). There was no correlation in *SIRT1/SIRT2* expression in neither the patients nor the controls (r_p_ < 0.3). Of note, *SIRT1* mRNA levels were lower in all the HIV patients regardless of their current cART (received for at least 6 months) (Figure 1d). Moreover, the lower expression of *SIRT1* in the HIV patients was corroborated at the protein level (Figure 1e). Histones are among the main targets of the deacetylating activity of SIRT1. We found that the HIV patients had higher levels of the acetylated form of histone H3 (H3K9) (Figure 1f), which is in line with the lower SIRT1 expression found in these individuals. Next, we analyzed *SIRT1* expression in relation to a variety of parameters in HIV patients (anthropometric factors, HIV-associated parameters and comorbidities) as well as plasma biochemical variables (lipids, glucose, transaminases, ferritin and CRP). These analyses included (i) non-continuous variables (such as gender, the presence of AIDS in the past, presence of comorbidities, e.g., diabetes mellitus, and biochemical status, e.g., hyperglycemia vs. normoglycemia, taking into account the normal adult reference range for blood glucose levels) and (ii) continuous variables (e.g., all the biochemical analyses that implicated a range of values). This implies that some parameters (e.g., blood glucose levels) were analyzed by two different approaches: as categories and as a continuous range of values. There was no significant difference in the expression of *SIRT1* regarding any of the non-continuous variables (Supplementary Figure S1), while in the case of the continuous variables, weak but statistically significant correlations were detected between *SIRT1* expression and total cholesterol, LDL level and number of leukocytes (r_p_ = 0.308, *p* = 0.011; r_p_ = 0.310, *p* = 0.010 and r_p_ = 0.354, *p* = 0.003, respectively). Of note, a negative correlation was observed between SIRT1 level and HIV viral load (r_s_ = −0.267, *p* = 0.029). Interestingly, we also observed a tendency towards higher levels of *SIRT1* with increased age and BMI in the uninfected controls (r_p_ = 0.362, *p* = 0.019; r_p_ = 0.428, *p* = 0.023), but not in the HIV patients (r_s_ = 0.167, *p* = 0.175; r_s_ = 0.192, *p* = 0.122).

The main cell populations within the PBMCs are lymphocytes (70–90% T cells, B cells and NK cells) and monocytes (10–20%). Given that HIV patients have an altered T cell CD4/CD8 ratio, shown in our previous study with a similar cohort [12], we analyzed whether the different cell type proportions within the PBMCs accounted for the observed diminished expression of SIRT1. We previously reported [12] that HIV patients showed no significant differences in the expression of *CD14* and *CD19*, markers of monocytes and B lymphocytes, respectively. Here, we evaluated the correlation of SIRT1 expression with *CD4*, *CD8* (*CD8A*, one of the two genes for CD8), *CD14* and *CD19* levels (Table 4). *SIRT1/CD4* and *SIRT1/CD14* did not show a correlation in either the HIV patients or the controls. Interestingly, the uninfected individuals exhibited moderate positive *SIRT1/CD8A* and *SIRT1/CD19* correlations (r_p_ = 0.634, *p* < 0.0001 and r_p_ = 0.599, *p* < 0.0001, respectively), while these correlations were weak in the HIV patients (r_p_ < 0.4). In addition, while in the case of the *SIRT1/CD8A* correlation, the cART regimen that the patients were taking had no effect, individuals exposed to integrase inhibitor-containing therapies presented an *SIRT1/CD19* correlation coefficient (r_p_ = 0.5148, *p* < 0.05) that was similar to that obtained for the uninfected controls (Table 5). Whether these correlations between SIRT1 and specific leukocyte markers are causal needs to be further explored.

## 4. Discussion

Although SIRT1 is recognized as cytoprotective in many chronic inflammatory and age-related diseases, its role in the chronic pathologies associated with treated HIV infection has not been studied in detail. Of note, HIV-1 Tat protein, which itself is a substrate of the deacetylase activity of SIRT1, blocks the ability of SIRT1 to deacetylate NF-κB, which may contribute to chronic immune activation and the pro-inflammatory state of HIV patients [14].

HIV patients in our cohort had a lower expression of *SIRT1* and this occurred under all three classes of antiretroviral drugs (NNRTI, PI and II), suggesting that it was related to the underlying (inflammatory) condition of otherwise well-controlled HIV patients. Additionally, this effect was specific to *SIRT1* as we detected no alterations in two other sirtuins, *SIRT2* and *SIRT3*. Our results support previously published data indicating diminished *SIRT1* mRNA in CD4^+^ cells from HIV patients [15]; however, the paper in question offers no information about their comorbidities, HIV-related parameters or the antiretroviral treatment they received. In another study, CD4^+^ cells from HIV patients under cART displayed a significantly higher expression of SIRT1 in comparison with non-controllers [16], and this may have been the result of the lower presence of Tat due to the effective therapy. Of note, evidence from the general population points to a decline in SIRT1 activity with age, but several studies suggest that its abundance actually increases with age, as described in both animal models [17] and humans [18]. Our results are in line with these observations, as we have detected a tendency towards higher levels of SIRT1 with increased age in the uninfected controls but not in the HIV patients. The decrease in SIRT1 levels in HIV patients may be of clinical relevance for the pathogenesis of various comorbidities such as the activation of astrocytes in HIV-associated neurocognitive disorder [19] and the onset of diabetes-related chronic kidney disease [20]. Given that the diminished presence of SIRT1 leads to the increased acetylation and activation of p53 and p65 (NF-κB) and therefore the enhanced expression of senescence and inflammatory markers, respectively, the pathogenic potential of this phenomenon is far reaching and could underly many pathologies such as cardiovascular and chronic liver diseases. The importance of sirtuins for the development of HIV-associated comorbidities has been the subject of a recent review [21].

Accumulating evidence points to the fundamental role of SIRT1 as a regulator of both the innate and the adaptive immune responses. For instance, it plays crucial roles in CD8^+^ T cell differentiation and function [9]. We observed a moderate but significant *SIRT1/CD8A* correlation in the controls, which was weaker in the HIV patients, and given that CD8^+^ T cells are fundamental in HIV infections, this finding may be highly relevant for HIV patients. A similar result was obtained for *SIRT1/CD19* (marker of B lymphocytes). SIRT1 is important for B cells and in the context of HIV, B cell hyperactivity and dysfunction have been reported in some organs in HIV-infected individuals (i.e., intestine), with the possible involvement of the miR-34a–SIRT1–p65 pathway [22]. Additionally, activated B cells express low SIRT1 protein levels [23] and SIRT1 seems to increase B cell survival [24]. Finally, the differences detected between the HIV patients and the uninfected individuals regarding the correlations of *SIRT1* and these specific leukocyte subpopulation markers need to be further confirmed and analyzed through functional studies as these differences may be of relevance for the understanding of HIV infection and the development of its comorbidities.

## 5. Conclusions

The PBMCs obtained from HIV patients had a lower expression of SIRT1, an effect intrinsically linked to the presence of the HIV infection in these individuals as no correlation was found with the patients’ characteristics, variables and cART regimens. The pathophysiological relevance of this finding may be linked to the complex pathogenesis in this population. Also, HIV-patients exhibit altered correlation of SIRT1 and certain lymphocyte subpopulations markers, namely CD8^+^ T and B cells.

## Figures and Tables

**Table 1:** Pairs of primers used for quantitative RT-PCR experiments. The name of the gene, forward and reverse sequences, and size of the product (base pairs) are shown.

Genes	Gene Symbol	Forward (F) and Reverse (R) Primers 5′-3′	Size (bp)
Sirtuins	*SIRT1*	F: TGGGTACCGAGATAACCTTCT R: TGTTCGAGGATCTGTGCCAA	181
	*SIRT2*	F: CATCCCCGACTTTCGCTCTC R: ATGGTTGGCTTGAACTGCCC	165
	*SIRT3*	F: TCTGCCACCTGCACAGTCTGC R: CAGCGGCTCCCCAAAGAACAC	138
Lymphocyte markers	*CD4*	F: TTCCCAGAAGAAGAGCATACAA R: TGGCAGTCAATCCGAACAC	254
	*CD8A*	F: CCCTTTACTGCAACCACAGG R: GGAAGGACTTGCTCCCTCAA	167
	*CD19*	F: AGCGAATGACTGACCCCACC R: AGCCCTCCCCTTCCTCTTCT	255
	*CD14*	F: CCCGAGTCAACAGGGCATT R: GATGTTTCAGGGAGGGGGAC	121
Housekeeping gene	*GAPDH*	F: CTTCTTTTGCGTCGCCAGCC R: TTCTCAGCCTTGACGGTGCC	232

**Table 2:** Characteristics of the HIV patients. Information about the patients included general characteristics (age and BMI), clinical data for other diseases and common biochemical and immunological blood test results, while alcohol consumption, drug use and cigarette smoking were self-reported. The Charlson comorbidity index (CCI), which predicts 10-year survival, was calculated depending on the number and severity of the present pathologies—the higher the score, the greater the comorbidity. For cardiovascular risk (CVR), the patients were stratified in high-risk (patients with previous CV disease, diabetes mellitus, more than one risk factor and a Framingham calculated coronary risk for 10 years >20%), moderate-risk (more than one risk factor and a Framingham coronary risk for 10 years <20%), low-risk (one risk factor) and zero-risk groups. CVR factors include hypertriglyceridemia, hypercholesterolemia, hypertension, a high BMI, a family background of CV disease and a diagnosis of heart ischemia. HIV-related factors were also considered, including mode of transmission, years since diagnosis, changes in the regimen of antiretroviral therapy (called antiretroviral therapy line or how many times the therapy has been changed for the same patient) and previous development of AIDS—defined by internationally accepted criteria, including the presence of an AIDS-defining condition or having a CD4^+^ cell count lower than 200 cells/mm^3^ regardless of the existence of an AIDS-defining condition. Values are expressed as number of patients and percentage of total number of patients for non-numerical data, and median (1st and 3rd percentiles) for numerical data, which were all non-parametric.

General Characteristics of the HIV Patients		
**gender**	male	56 (80%)
	female	14 (20%)
**age (years) median (IQR)**	52.00 (45.75–56.00)	
**BMI (kg/m^2^) median (IQR)**	24.90 (23.33–27.87)	
**smoking**	yes	25 (35.72%)
	no	33 (47.14%)
	previous smoker	12 (17.14%)
**alcohol use**	yes	1 (1%)
	no	69 (99%)
**Comorbidity-Related Parameters**		
**HCV coinfection**	yes	20 (28.57%)
	no	48 (68.57%)
	no data	2 (2.86%)
**HBV coinfection**	yes	2 (2.86%)
	no	63 (90.00%)
	no data	5 (7.14%)
**diabetes mellitus**	yes	15 (21.43%)
	no	55 (78.57%)
**dyslipidemia**	yes	22 (31.43%)
	no	48 (68.57%)
**cardiovascular risk**	none	16 (22.86%)
	low	16 (22.86%)
	medium	20 (28.57%)
	high	16 (22.86%)
	no data	2 (2.86%)
**Charlson index median (IQR)**	2.00 (0.00–6.00)	
**HIV-Related Parameters**		
**years with HIV median (IQR)**	19 (8.50–27.00)	
**years with antiretroviral therapy median (IQR)**	15 (4.00–21.25)	
**mode of transmission**	HO/BI	25 (35.71%)
	HTSX	17 (24.29%)
	IDU	21 (30.00%)
	other	7 (10.00%)
**AIDS**	yes	18 (25.71%)
	no	52 (74.29%)
**antiretroviral therapy (in addition to NRTI backbone)**	NNRTIs	11 (15.71%)
	PIs	16 (22.86%)
	IIs	28 (40%)
	combination	15 (21.43%)
**antiretroviral therapy line median (IQR)**	5 (2.00–8.25)	
**max viral load (copies/mL) median (IQR)**	79,500 (5925–361,000)	
**CD4 nadir count (cell/mL) median (IQR)**	207.5 (71.50–339.00)	
**CD4:CD8 median (IQR)**	0.730 (0.478–1.080)	

BMI, body mass index; HBV, hepatitis virus B; HCV, hepatitis virus C; HO/BI, homosexual/bisexual; HTSX, heterosexual; IDU, injection drug user; IIs, integrase inhibitors; NRTIs, nucleoside reverse transcriptase inhibitors; NNRTIs, non-nucleoside reverse transcriptase inhibitors; PIs, protease inhibitors.

**Table 3:** Characteristics of the control subjects. Information about the controls included general characteristics (gender, age and BMI), while alcohol consumption and cigarette smoking were self-reported. Values are expressed as number of control individuals and percentage of the total number of control individuals for non-numerical data, and median (1st and 3rd percentiles) for numerical data, which were all non-parametric.

Characteristics of the Uninfected Control Population		
**gender**	male	female
	33 (76.74%)	10 (23.26%)
**age (years) median (IQR)**	47 (42.00–54.25)	
**BMI (kg/m^2^) median (IQR)**	26.10 (24.01–28.32)	
**smoking**	yes	no
	10 (23.26%)	33 (76.74%)
**alcohol consumption**	yes	no
	33 (76.74%)	10 (23.26%)

BMI, body mass index.

**Table 4:** Correlation between the expression of *SIRT1* and specific markers of PBMC subpopulations in the HIV patients and controls. Correlation coefficients were calculated to assess the strength and direction of the linear relationships between pairs of variables. Correlations are expressed as Pearson’s correlation coefficient (r_p_) when both variables were normally distributed (parametric data) and Spearman’s correlation coefficient (r_s_) for non-parametric data. Variable pairs with correlation coefficients >0.3 were considered correlated and are displayed in bold. Statistical significance is also shown. *n* = 42–43 for the control group and *n* = 65–69 for the HIV^+^ group.

Markers of PBMC Subpopulations		
	Control	HIV^+^
***CD4***	r_s_ = 0.186	r_s_ = 0.295
	*p* = 0.231	*p* = 0.017
***CD8A***	**r_p_ = 0.634**	r_p_ = 0.342
	***p* < 0.0001**	*p* = 0.005
***CD14***	r_s_ = 0.131	r_p_ = −0.018
	*p* = 0.408	*p* = 0.888
***CD19***	**r_p_ = 0.599**	r_p_ = 0.340
	***p* < 0.0001**	*p* = 0.006

**Table 5:** Correlation between the expression of SIRT1 and specific markers of PBMC subpopulations in HIV patients in relation to their cART. Correlation coefficients were calculated to assess the strength and direction of the linear relationships between pairs of variables. Correlations are expressed as Pearson’s correlation coefficient (r_p_) when both variables were normally distributed (parametric data) and Spearman’s correlation coefficient (r_s_) for non-parametric data. Variable pairs with correlation coefficients >0.3 are considered correlated and are displayed in bold. Statistical significance is also shown. *n* = 11 for NNRTIs (nucleoside reverse transcriptase inhibitors), *n* = 14–15 for PIs (protease inhibitors) and *n* = 23 for IIs (integrase inhibitors).

Drug Family	*SIRT1* vs. *CD4*	*SIRT1* vs. *CD8A*	*SIRT1* vs. *CD14*	*SIRT1* vs. *CD19*
**NNRTIs**	r_s_ = 0.527	r_p_ = 0.168	r_p_ = −0.342	r_p_ = 0.329
	*p* = 0.100	*p* = 0.622	*p* = 0.303	*p* = 0.323
**PIs**	r_s_ = 0.024	r_s_ = 0.475	r_p_ = −0.416	r_p_ = −0.018
	*p* = 0.940	*p* = 0.076	*p* = 0.139	*p* = 0.951
**IIs**	r_s_ = 0.157	r_p_ = 0.336	r_p_ = −0.276	**r_p_ = 0.515**
	*p* = 0.474	*p* = 0.117	*p* = 0.203	***p* = 0.012**

## Data Availability

Not applicable.

## Supplementary Materials

The following are available online at https://www.mdpi.com/article/10.3390/cells11030348/s1, Figure S1: mRNA levels of *SIRT1* in the PBMCs of the HIV patients in relation to different anthropometric measurements (BMI, gender and age), HIV-associated parameters (development of AIDS, source of infection and duration of antiretroviral treatment), the presence of co-infections, comorbidity indicators and blood biochemical parameters.
