# Supplementary figures and images for: Down-Regulation of the Longevity-Associated Protein SIRT1 in Peripheral Blood Mononuclear Cells of Treated HIV Patients
Source: Cells. 2022 Jan 20;11(3):348. doi: 10.3390/cells11030348 (PMC8834054; doi:10.3390/cells11030348)

Supplementary Figure S1

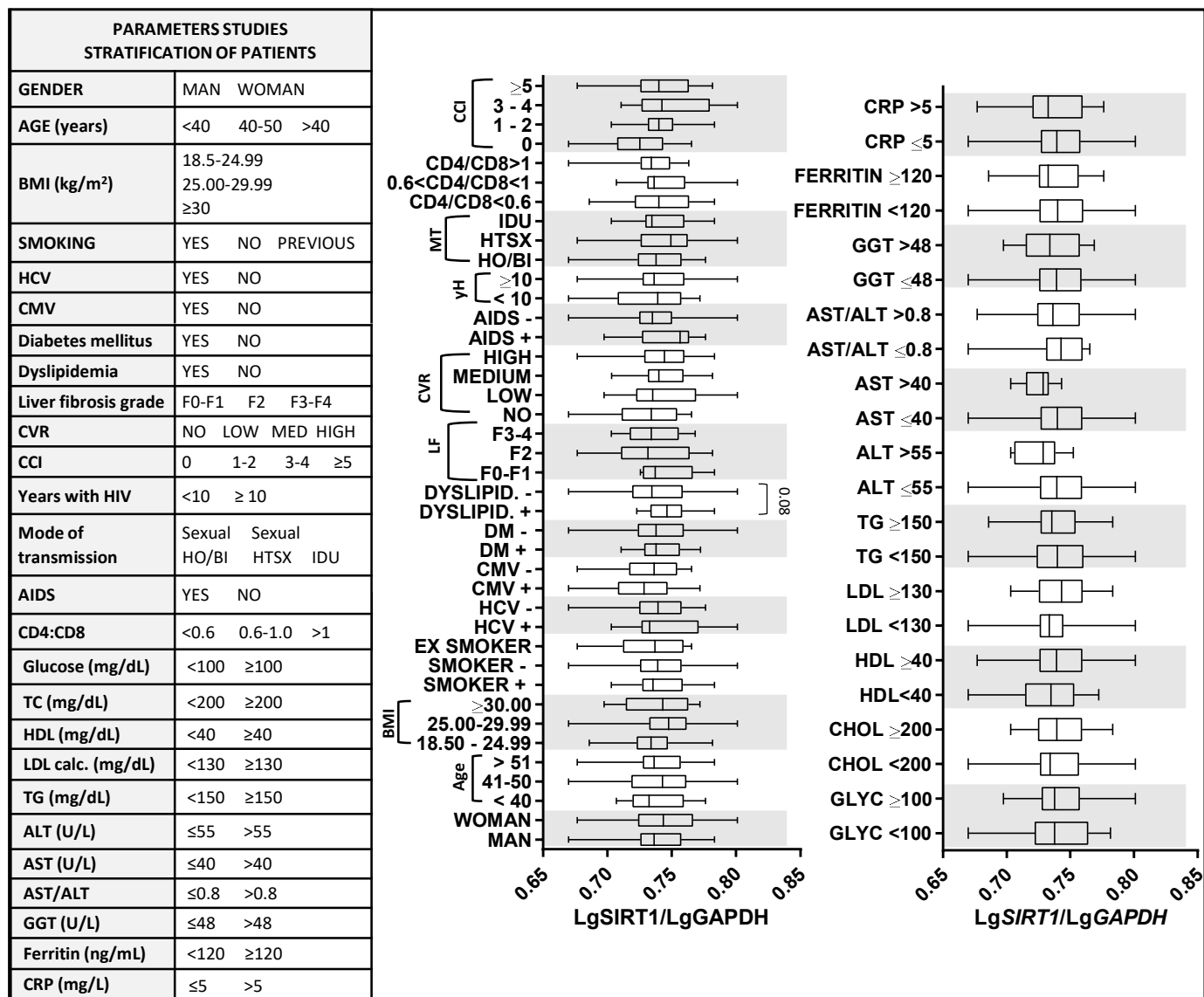

Supplement: Supplementary file 1 [file cells-11-00348-s001.zip › cells-1446044-supplementary.pdf]
